# Expression and localization of forkhead transcriptional factor 2 (Foxl2) in the gonads of protogynous wrasse, *Halichoeres trimaculatus*

**DOI:** 10.1186/2042-6410-1-3

**Published:** 2010-11-04

**Authors:** Yasuhisa Kobayashi, Ryo Horiguchi, Ryo Nozu, Masaru Nakamura

**Affiliations:** 1 Sesoko Station, Tropical Biosphere Research Center, University of the Ryukyus, 3422 Sesoko, Motobu 905-0227, Okinawa, Japan; 2 Solution-Oriented Research for Science and Technology (SORST), Kawaguchi, Saitama, Japan

## Abstract

**Background:**

Three-spot wrasse, *Halichoeres trimaculatus*, is a marine protogynous hermaphrodite fish. Individuals mature either as initial phase (IP) males or females. Appropriate social cues induce the sex change from IP female to terminal phase (TP) male. However, the molecular mechanisms behind such a sex change remain largely unknown. Recently, the forkhead transcription factor 2 (Foxl2) was identified as an essential regulator of vertebrate ovarian development/function/phenotype. Inspired by this information, we characterized the expression patterns of Foxl2 in the protogynous wrasse assuming Foxl2 as the female-specific marker in this species.

**Methods:**

First, we clonedFoxl2 cDNA from ovary by reverse transcription polymerase chain reaction (RT-PCR) followed by rapid amplification of cDNA ends (RACE). Next, we analysed expression pattern of Foxl2 messenger RNA (mRNA) and protein in gonads of different sexual phases by real time quantitative PCR assay and flour fluorescence immunohistochemical method, respectively. Additionally, we studied the changes in Foxl2 expression pattern during aromatase inhibitor (AI)-induced sex change.

**Results:**

The amino acid sequence (306 AA) of wrasse Foxl2, especially the forkhead domain, shows high identity with that of other reported teleost Foxl2s. Quite unexpectedly, no sexual dimorphism was observable between the testes and ovary in the expression pattern of Foxl2. In female phase fish, signals for Foxl2 protein were detectable in the granulosa cells, but not the theca cells. Transcript levels of Foxl2 in the testes of IP and TP males were identical to that in the ovaries of females and, further, Foxl2 protein was found to be localized in the interstitial cells including tubules and Leydig cells. Treatment with AI induced sex change in male gonads and an up-regulation was seen in the expression of Foxl2 in these gonads.

**Conclusions:**

Unlike in other vertebrates, including teleosts, Foxl2 may have a different role in the naturally sex changing fishes.

## Introduction

Similar to mammals, most of the teleosts are gonochorists; individuals develop either as males or females and remain the same sex throughout their lives [1]. However, there are many hermaphroditic fishes that live in the tropical and subtropical sea areas [2]. They change their sex from female to male (protogynous) or vice versa (protandrous) or both-directions (serial-sex change) [1,2]. Although numerous studies have described the endocrinological aspects of sex change in the recent past, knowledge of the general mechanisms that mediate sex change is still lacking.

Three-spot wrasse, *H. trimaculatus*, is one of the protogynous hermaphrodite species [3]. Individuals of this species mature initially either as males or females. Under the appropriate social conditions, the initial phase (IP) males and females change to terminal phase (TP) males. IP males change only their reproductive status, while IP females change their sexuality and the gonadal phenotype. We used this species as an experimental animal model in order to elucidate the physiological mechanism behind the sex-change as they are abundantly available in Okinawa, Japan, and they can easily be captured and handled.

Studies have shown that estrogens (estradiol-17β: E2) are the key factors in sex change [4]. In protogynous saddleback wrasse, a rapid decline in the levels of serum oestrogen was observed before the onset of gonadal sex change [5]. In three-spot wrasse, blocking oestrogen synthesis using aromatase inhibitor (AI) induced a complete female-to-male sex change [6,7] and E2 treatments on IP male induced the sex change in the reverse direction (male-to-female) [8]. Similarly, in the protandrous black porgy, E2 treatments induced male-to-female sex change [9] and the use of AI blocked the process of sex change in this species [10]. Cytochrome P450 aromatase (P450arom) is the key enzyme that catalyzes the conversion of androgen to oestrogen [11]. Therefore, in order to understand the detailed molecular mechanisms that precede sex change it is necessary to examine the factors or genes involved in the regulation of aromatase expression.

Forkhead transcriptional factor 2 (Foxl2, gene; *Foxl2*) is a member of the winged helix/forkhead group of proteins [12]. Recently there have number of ontogenic expression studies on Foxl2 have been carried out in vertebrates [12,13]. These studies revealed that Foxl2 is one of the earliest markers of ovarian differentiation in vertebrates [14]. As in mammals, aromatase and Foxl2 were found to be co-localized in the adult ovaries of medaka [15], tilapia [16] and flounder [17] and also in the immature gonads of these species just after initiation of ovarian differentiation [18,19]. In tilapia, Foxl2 directly binds to the promoter region of the cytochrome P450 aromatase (P450arom) gene, resulting in the activation of P450arom transcription [20]. Although studies on the roles of Foxl2 in sex determination and differentiation among gonochoristic teleosts are increasing, there is only a limited amount of information available for Foxl2 in hermaphrodite fishes.

In this study, in order to find clues to the role of Foxl2 in protogynous hermaphrodite three-spot wrasse, we cloned wrasse Foxl2 complimentary DNA (cDNA) and analysed its expression patterns in gonads by real time quantitative polymerase chain reaction (rtq PCR) assay and fluorescence immunohistochemistry. We also examined the changes in Foxl2 expression in the gonads undergoing sex-change in response to AI treatment.

## Methods

### Experimental fishes and sample collection

Adult three-spot wrasses were collected by roll net from Sesoko Beach (Okinawa Prefecture, Japan) and then maintained in 500-L tanks with flow-through seawater at the Sesoko Station (University of the Ryukyus). The sexes of the fish were determined by applying light pressure on the abdomen to elicit gamete release [7,8]. Fish which did not release sperm were regarded as females. TP males were distinguished from IP males on the basis of body colouration. All the fish were anesthetized with 2-phenoxyethanol (Wako Chemicals, Osaka, Japan) before sampling. After measuring the total length and body weight the fish were decapitated. Brain, pituitary, eye, intestine, gill, muscle, liver, spleen and pieces of the gonads (24.96 ± 4.23 mg) were dissected out and stored in an RNAlater reagent (Ambion, TX, USA) at -30°C until the RNA was extracted. The remainder of the gonads were fixed with 4% paraformaldehyde (PFA) for histological analysis. All animal handling and experiments were conducted in accordance with our Guide for Care and Use of Laboratory Animals (Doubutu-jikken-kisoku, 19.6.26) approved by the University of the Ryukyus.

### RNA extraction and isolation of wrasse *Foxl2 *cDNA

The total RNA was extracted using the RNeasy mini kit with RNase-free DNase kit (Qiagen, Venlo, the Netherlands) according to the manufacturer's protocols. The RNA concentration was measured using a NanoDrop spectrometer. RNA samples were used for cloning and rtq PCR assay.

One microgram of total RNA from the ovary was reverse-transcribed using an oligo (dT) primer and Superscript II reverse transcriptase (Invitrogen, CA, USA) according to the manufacturer's instructions. RT-PCR was performed using degenerate primers (dn Foxl2-F1: gagaagmgbctyacgctgtccgg, dn Foxl2-R1: cccartawgagcartgcatcat) designed from the conserved regions of known Foxl2 sequences from other vertebrates. The amplicon was T-A ligated into pGEM-T-Easy vector (Promega, WI, USA) and sequenced. Based on the sequence information of the partial complimentary DNA (cDNA) of Foxl2, rapid amplification of cDNA ends (RACE) procedures were performed in order to isolate the 5' and 3' ends of the cDNA (SMART cDNA library construction Kit; Takara, Shiga, Japan). The initial PCR amplification in each RACE procedure was followed by a nested PCR. Gene-specific primer (GSP) sets (5' RACE: 5GSP1; accaggagttgttcatgaagctggact, 5GSP2; tagttccccttctcaaacatgtcctca, 3'RACE: 3GSP1; agtccagcttcatgaacaactcctggtcg, 3GSP2; atccccaccatgcccagcagctgagcccg) were used in combination with adaptor primers. Finally, the open reading frame (ORF) of the wrasse Foxl2 was generated from ovarian RNA by RT-PCR with primers targeting the untranslated regions immediately upstream (actagcatttggactggagttg) and downstream (ttttgaaatcacaggaatcag) to the ORF. The final PCR product was subcloned and sequenced in both directions. The PCR cycle conditions were 30 cycles, with 94°C for 15 s, 50°C for 30 s and 72°C for 30 s.

**Figure 1:**
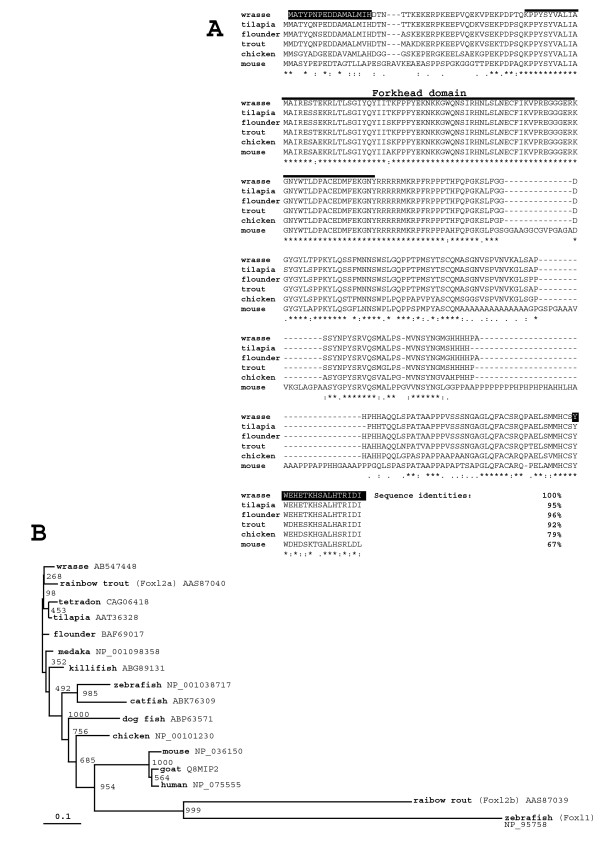
**Sequence and phylogenetic analysis of Foxl2 proteins**. (A) Sequence alignment of Foxl2 proteins. Forkhead domain is underlined. The boxed peptide sequences were used for the synthesis of antigens of wrasse Foxl2. The identification of wrasse Foxl2 sequence with other sequences is indicated in percentage against each sequence. Amino acids identical to those in other species are indicated by asterisks (*). Colon (:) indicates conserved substitution and dot (.) indicates semi-conserved substitution. (B) A phylogenetic tree comprising the subfamily of Foxl2 proteins was constructed using neighbour-joining method. GenBank Accession Numbers for the aligned proteins are presented with species name. The tree was rooted using zebrafish Foxl1 as outgroup. The values are bootstrap probabilities estimated by 1000 replications. Horizontal line indicates genetic distance.

Alignment of the amino acid sequences of Foxl2 from different species was performed using the ClustalW sequence alignment program. Homology value (percentage of amino acid sequence identity) was calculated by pair wise alignment. The phylogenetic tree was constructed using neighbour-joining (NJ) method [21] and viewed with TreeViewX (Version 0.5.0). Details of the program settings are given in the legend for Figure 1.

### Quantification of wrasse *Foxl2 *mRNA expression

Wrasse *Foxl2 *transcripts in gonads and other tissues were determined by rtq PCR assay. Samples were reverse transcribed from 500 ng of total RNA in a 20-μL reaction volume using random primers and Ominiscript reverse transcriptase (Qiagen). A 5 ng dose of cDNA was used for rtq PCR. Assays (in triplicate) were repeated at least twice. Primer pairs were designed using Primer3 software [22]. rtq-PCR reactions were performed using SYBR Premix ExTaq (Takara) on the ABI Prism^® ^7000 Sequence Detection System (Applied Biosystems, PE Applied Biosystems, CA, USA). The data of rtq PCR were analysed using ABI prism 7000 SDS software (version 1.1). The standard copy number of Foxl2 gene was estimated based on molecular weight of plasmid ligated to wrasse Foxl2 ORF region. The copy number of wrasse Foxl2 transcripts per total RNA in gonads was calibrated by gonad weight. Relative changes in Foxl2 expression in extra-gonadal tissues were determined using 2^-^^∆^^∆^^Ct ^method [23] with the constitutive elongation factor 1 α (ef1a) as a normalizing control. The following primers were applied for rtq PCR: Foxl2 forward, 5'-aagaggagccggtccaggagaa-3'; Foxl2 reverse, 5'-gctctcccggatggccatgg-3'; ef1a forward, 5'-aagggagccgatcacttcaa-3; and ef1a reverse, 5'-aatccagcacaggtgcgtaa-3'.

### Antibody production

The antigen of wrasse Foxl2 was two oligo-peptides corresponding to the wrasse Foxl2 amino acid sequences; MMATYPNPEDDAMALMIC and YWEHETKHSALHTRIDI which are situated on N- and C-terminus respectively (Figure 1). After purification of the synthesized oligo-peptides, Japanese white rabbit (specific pathogen-free) was immunized with these antigens (six times weekly).

### Localization of wrasse Foxl2 protein in gonads by fluorescence immunohistochemistry

After removal of paraffin and dehydration, the sections of gonads were washed with phosphate buffered saline. The sections were treated with 10% normal goat serum for 10 min to block non-specific binding. Sections were then incubated with the antibody (1:2000 dilution) for overnight at 4°C. Primary antibody was detected using donkey anti-rabbit-IgG-Alexa 568 (Invitrogen) and DAPI was used to visualize the nuclei of the cells. As a control, sections were treated with antigen-absorbed primary antibody instead of primary antibody.

### Aromatase inhibitor treatments

In order to obtain animals in a transitional phase of sex change, we induced artificial sex change by AI in accordance with previously described methods [7]. Briefly, the females were placed in the same aquarium with one dominant TP male, which would inhibit natural sex change. Fadrozole (nonsteroidal AI; Novartis Pharma Inc, Tokyo, Japan) was used as AI in this study. It was dissolved in ethanol and mixed with the fish diet at the dose of 200 μg/g. AI treatment group fishes were fed with this diet twice daily for 2 weeks. The control group was fed a normal diet. The AI treatment experiment was replicated twice. After the AI treatment, gonads were removed and fixed in 4% PFA. Sectioned gonads were examined under a microscope and classified into one of six stages, the details of which are given elsewhere [24,25].

Stage I: normal female ovary. The fish of this stage had a gonad containing many mature vitellogenic oocytes and previtellogenic oocytes. No testicular tissues were present.

Stage II: degeneration of vitellogenic oocytes. This was a typical regressed ovary possessing degenerating oocytes (DO).

Stage III: degeneration of previtellogenic oocytes. Previtellogenic oocytes at the peri-nucleolus stage began to degenerate following the degeneration of vitellogenic oocytes.

Stage IV: proliferation of Leydig cells and presumptive spermatogonia. At this stage, a few DO and cysts of oocytes at the meiotic prophase still remained in the lamellae. In contrast to the decreased numbers of oocytes, there was an increase in number of cells presumed to be spermatogonia on the periphery of the lamellae.

Stage V: onset of spermatogenesis. Numbers of spermatogonia increased rapidly in the lamellae.

Stage VI: testes just after sex change. Sex change from female to male was completed by this stage.

### Statistical analysis

One-way ANOVA was used to compare mean values, followed by Turkey-Kramer Multiple comparison test using PRISM 5.0b software (GraphPad, CA, USA). The results are presented as means ± standard error of mean.

## Results

### Isolation of wrasse *Foxl2 *and sequence analysis

The isolated wrasse Foxl2 cDNA was 1989-bp long and encoded 306-amino acid proteins. The sequence of wrasse Foxl2 was submitted to the GenBank (accession No. AB547448). Figure 1A shows wrasse Foxl2 alignment with other reported Foxl2 of tilapia, flounder, trout, chicken and mouse. Wrasse Foxl2 was more similar to the fishes, especially those belonging to Perciformes order (over 90%), than other vertebrates. Figure 1A further reveals that Forkhead domain (FH), also known as 'winged helix' (the family of transcription factor domains), was highly conserved among vertebrates. Phylogenetic tree of Foxl2 was constructed using zebrafish Foxl1 as outgroup (Figure 1B). Vertebrate Foxl2 sequences except for rainbow trout Foxl2b shared high bootstrap values.

### Quantitative analysis of wrasse *Foxl2 *in different tissues

**Figure 2:**
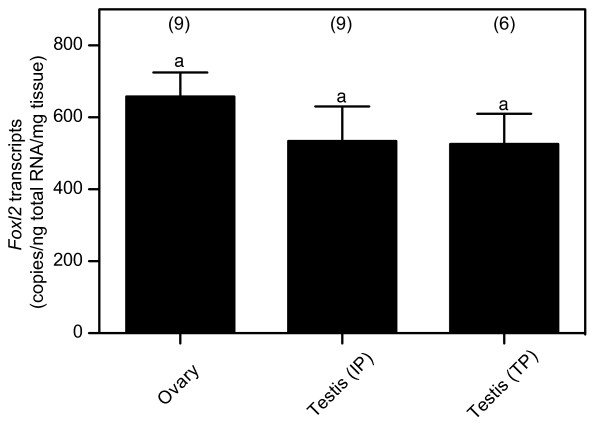
**Expression of *Foxl2 *transcripts in the ovary of female and testes of initial phase (IP) or terminal phase (TP) male**. The copy number of *Foxl2 *was calibrated by tissue weight. Each bar represents mean ± standard error of mean, as determined by a real time quantitative polymerase chain reaction assay. Numbers in parentheses indicated sample size. There were no significant difference noticed between groups.

**Figure 3:**
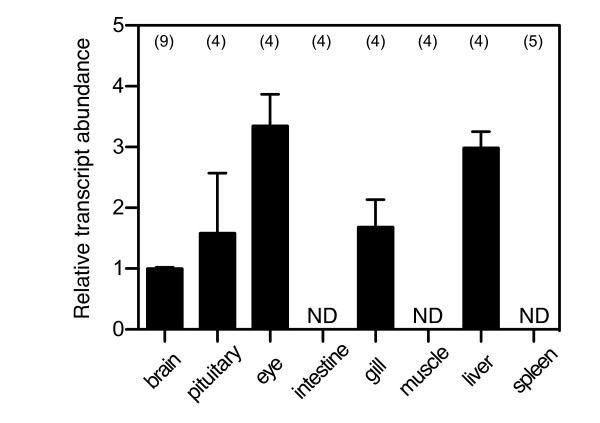
**Tissue distribution of *Foxl2 *in three-spot wrasse tissues**. Each bar represents mean ± standard error of mean, as determined by a real time quantitative polymerase chain reaction PCR assay. Values were normalized with a reference gene, *ef1a*. The transcript level is expressed relative to that of the brain sample, which represents the lowest detectable expression level. ND: transcripts were non-detectable. Numbers in parentheses indicated above the bars represent sample size.

In order to determine whether or not there was any sex difference in *Foxl2 *expression, we first quantified the levels of wrasse *Foxl2 *transcripts in the gonads of different sexual phases using rtq PCR assay (Figure 2). *Foxl2 *was expressed not only in the ovary but also IP and TP testes. The highest expression of *Foxl2 *was observed in the ovary of the female. However, no significant difference could be observed between ovary and testes of different reproductive phases. Transcripts of wrasse *Foxl2 *were found in the brain, pituitary, eye, gill and liver and, among these, eye and liver showed high levels of transcripts in addition to the gonads. We could not detect *Foxl2 *transcripts in intestine, muscle and spleen (Figure 3). Expression levels in these organs were approximately 10-fold lower than that in the gonads (data not shown). Sexual dimorphism was not detected in the expression levels of *Foxl2 *in tissues other than the gonads.

### Localization of Foxl2 protein in the gonads

**Figure 4:**
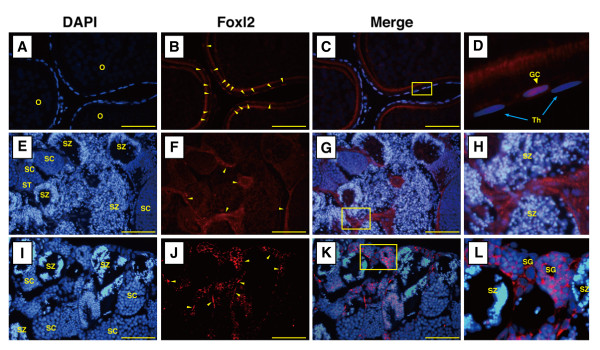
**Localization of Foxl2 protein by immunofluorescence in three-spot wrasse gonad sections**. Red represents Foxl2 protein, whereas blue represents the nucleus. (A-D) Section of an ovary of a female fish. (D) High magnification of the area inside the rectangle shown in (C). The arrowhead indicates a positive signal in granulosa cell (GC) and an arrow indicates theca cells (Th) that have no signals. (E-H) Sections of the testis of an initial phase male. (H) High magnification of the area inside the rectangle shown in (G). (I-L) Sections of the testis of a terminal phase male. (L) High magnification of the area inside the rectangle shown in (K). O: oocyte; GC: granulosa cell; Th: Theca cell; SG: spermatogonia; SC: spermatocyte; ST: spermaid; SZ: spermatozoa. Bar = 50 μm.

In order to examine cellular location of Foxl2 expression in the wrasse gonads, fluorescence immunohistochemical observations were carried out using mature ovary and IP and TP testes with specific antibody for wrasse Foxl2 protein (Figure 4). In the ovary, Foxl2 immunoreactivity was confined exclusively to the granulosa cells but not the theca cell or the oocyte (Figure 4A-D). In testes of IP and TP males, specific signals were observed in interstitial cells (Figure 4E-L).

### Changes in *Foxl2 *mRNA expression during AI induced-sex change

**Figure 5:**
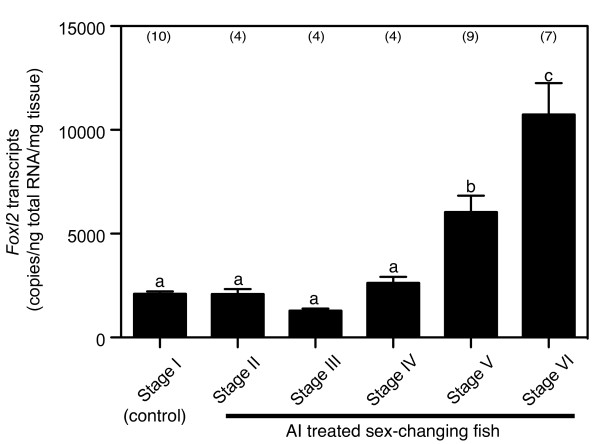
**Changes in *Foxl2 *messenger RNA levels in the sex-changing gonads induced by aromatase inhibitor (AI) treatment**. The copy number of *Foxl2 *was calibrated by tissue weight. Each bar represents mean ± standard error of mean as determined by a real time quantitative polymerase chain reaction assay. Gonadal stages of sex change are described in the Material and methods section of the article. Data points not sharing the same alphabets are shown to significantly different by Turkey-Kramer multiple comparison test. Numbers in parentheses indicated sample size.

In order to investigate changes in *Foxl2 *mRNA expression in the gonad during sex change, AI treatment was carried out by feeding adult females (*n *= 28) with a diet containing AI. Two weeks of AI treatment produced gonads in various stages of sex-change (sample number is mentioned in Figure 5). In contrast, all fishes in control groups remained as stage I females. Analysis of *Foxl2 *expression pattern in the gonads during sex-change by rtq PCR assay revealed that *Foxl2 *gradually increased depending on the stage of sex change (Figure 5).

### Changes in Foxl2 protein in the sex-changing gonads

**Figure 6:**
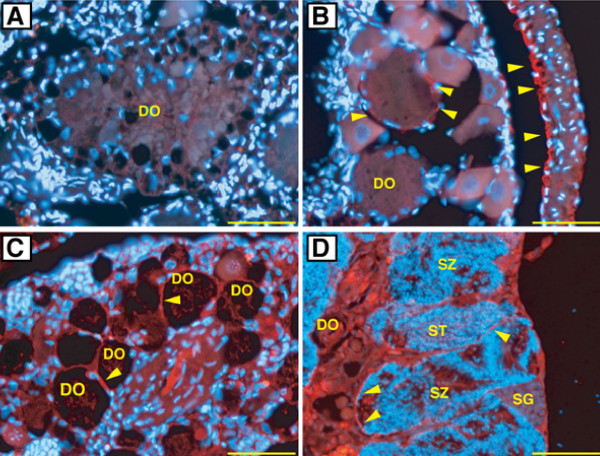
**Localization of Foxl2 protein by immunofluorescence in the sex-changing gonad of three-spot wrasse**. Red represents Foxl2 protein, whereas blue represents the nucleus. (A) Gonad at stage II. Degenerating oocyte (DO) is shown. (B) Section of gonad at stage III. (C) Section of gonad at stage IV. (D) Section of gonad at stage V. An arrowhead indicates a positive signal. Bar = 50 μm.

In the stage II gonad, we observed degenerating oocytes. Granulosa cells got separated from DO and migrated into the oocytes. However, we could not detect positive signals in these granulosa cells (Figure 6A). Some of the follicle cells around the oocytes showed signals for Foxl2 protein at stage II and III (Figure 6B). Strong signals were observed in the inner cell layer of the ovarian tunica (Figure 6B). At stage IV, positive signals were observed in the connective tissues near the DO (Figure 6C). Further, signals were detectable in cell layers around the cysts and interstitial cells in stage V gonad (Figure 6D).

## Discussion

It was previously reported that rainbow trout had two Foxl2 genes (namely, Foxl2a and Foxl2b) [26], expressed specifically in the ovary, but displaying different expression patterns during gonadal differentiation. However, we could find only a single copy of the Foxl2 gene from wrasse. We also examined the expression level of *Foxl2 *in the gonads belonging to different sexual phases. As expected, *Foxl2 *mRNA was expressed in the ovary and signals of Foxl2 protein were localized in granulosa cells, but not in the theca cells. In other teleosts, P450arom mRNA or protein is located in granulosa cells and Foxl2 has been shown to regulate the expression of P450arom gene [27-30]. Similarly, wrasse Foxl2 also might be involved in the regulation of P450arom expression in the ovary of the female, as in tilapia [20], flounder [17] and other vertebrates [18,31].

A number of studies of several vertebrates have reported a high expression of Foxl2 exclusively in the ovary [32]. Therefore, Foxl2 is, in general, considered to be a good marker for ovarian differentiation. However, expression of Foxl2 was reported in the adult testis of a few species such as catfish [33], rainbow trout [26], frog [19] and chicken [18] - although the levels of these expressions were significantly lower than that of the ovary. In stark contrast, no sex differences were observed in *Foxl2 *expression in the gonads of three-spot wrasse. Levels of *Foxl2 *expression in the testes of IP and TP males were almost equal to that of the ovaries in females and, furthermore, Foxl2 proteins were localized in the interstitial cells including tubules and Leydig cells in the testis. These results suggest that the role of Foxl2 in sex-changing wrasse can be quite different from other gonochoristic vertebrates and that Foxl2 is not useful as a marker for the detection of ovarian tissue in three-spot wrasse.

In addition, the tissue distribution analysis revealed that the brain, pituitary, eye, gill and liver expressed Foxl2 mRNA is similar to that seen in tilapia [16] and catfish [33], but the expression levels were lower than that of the gonads. Expressions of Foxl2 in the brain and pituitary indicate that Foxl2 might be involved in the brain-pituitary-gonadal axis. However, no information on the roles of Foxl2 in these tissues has so are been gathered by any study.

Administration of AI to the females induced sex-change successfully which was a similar result to that seen in our previous studies [6,7]. Inter-individual variation was high among the AI treatment group in terms of their responses towards the treatment (judged from differences in the stages of sex-change). Such a high variability might have resulted from the differences between the fish in their intake of the AI-mixed feed. Unexpectedly, we detected *Foxl2 *expression in gonads belonging to all stages of sex-change and the levels increased in accordance with the progression in testis development. Foxl2 protein signals were observable in the sex-changing gonads, similar to those observed in the localization of steroid producing cells in the saddleback wrasse [34]. These observations indicate that Foxl2 controls the steroid production in the sex-changing gonads. In contrast to our results, down-regulation of *Foxl2 *expression has been reported in sex-changing gonads of trout [26] and chicken [35] in response to AI treatment. Moreover, Foxl2 over expression in tilapia has caused testis development in females [20]. A recent report showed that inducible deletion of Foxl2 in the ovarian follicles adult mouse resulted in an immediate up-regulation of the testicular gene, Sox9, and trans-differentiation of granulosa cells into Sertoli cells [36]. These studies suggest that Foxl2 is essential for the maintenance of the ovarian phenotype. Nevertheless, these animals lack Foxl2 expression in testis. Therefore, up-regulation of Foxl2 in AI-induced gonads undergoing sex change in wrasse might indicate a negative feedback - that is, in response to the AI treatment there could be an up-regulation in the levels of Foxl2 expression to restore the levels of declining estrogens.

Since AI treatment induced complete sex change, endogenous oestrogen may not be required for normal sex change and testis development in three-spot wrasse. However, this study a high expression of Foxl2 was observed in the testis, indicating that oestrogen may have a more important role in the spermatogenesis of this species than previously thought. Further, no significant sexual dimorphism was seen in the expression of oestrogen receptor in the adult gonads of this species in the different sexual phases [3]. Furthermore, in other wrasse species (*Thalassoma duperrey*), plasma oestrogen levels were similar between stage II to VI [5,24]. Recent evidence obtained from different vertebrates indicates that oestrogen regulates spermatogonial cell renewal by acting on Sertoli cells in the testis [37]. When the results of our study and those of previous reports are taken into consideration, it has to be assumed that the role of oestrogen in spermatogenesis of this species may contradict all the current understandings about the involvement of oetsrogen in the spermatogenesis of other species.

Our previous study reported that AI-induced and normal testes were similar [7] and the present study also could not find any histological differences between the stage VI gonads of AI-induced males and normal males developed either by maturation of IP males or by sex reversal of IP females. Therefore, further studies which emphasise the subtle qualitative differences between the AI-induced and normal testes are required in order to justify the disparity seen by the present study in the levels of Foxl2 between these two categories of gonads.

## Conclusions

In conclusion, we have demonstrated the expression pattern of Foxl2 in protogynous wrasse. Foxl2 was expressed not only in the ovary, but also the testis. These results suggest that, unlike in other teleosts and vertebrates, Foxl2 might have a different role and function pertinent to natural sex changing fishes.

## Abbreviations

P450 arom: P450 aromatase; AI: aromatase inhibitor; c-DNA: complimentary DNA; DO: degenerating oocyte; Fox12: forkhead transcription factor 2; GSP: gene specific primer; IP: initial phase; NJ: neighbour joining; ORF: open reading frame; PCR: polymerase chain reaction; PFA: paraformaldehyde; rtq PCR: real time quantitative PCR; RACE: rapid amplification of cDNA ends; TP: terminal phase;

## Competing interests

The authors declare that they have no competing interests.

## Authors' contributions

The work presented here was carried out in collaboration between all the authors. YK and MN defined the research theme. All authors read and approved the final manuscript.
